# Casein Kinase 2 Inhibitor, CX-4945, Induces Apoptosis and Restores Blood-Brain Barrier Homeostasis in In Vitro and In Vivo Models of Glioblastoma

**DOI:** 10.3390/cancers16233936

**Published:** 2024-11-24

**Authors:** Valentina Bova, Deborah Mannino, Ayomide E. Salako, Emanuela Esposito, Alessia Filippone, Sarah A. Scuderi

**Affiliations:** 1 Department of Chemical, Biological, Pharmaceutical, Environmental Science, University of Messina, Viale Ferdinando Stagno d’Alcontres, 31, 98166 Messina, Italy; valentina.bova@unime.it (V.B.); deborah.mannino@unime.it (D.M.); ayomide.salako@studenti.unime.it (A.E.S.); eesposito@unime.it (E.E.); sarahadriana.scuderi@unime.it (S.A.S.); 2 Department of Statistics, Computer Science, Applications (DiSIA), University of Florence, 50121 Firenze, Italy

**Keywords:** casein kinase 2, glioblastoma, blood–brain barrier, inflammation

## Abstract

**Background:** In oncology, casein kinase 2 (CK2), a serine/threonine kinase, has a dual action, regulating cellular processes and acting as an oncogenic promoter. **Methods**: This study examined the effect of CX-4945, a selective CK2 inhibitor, in a human U-87 glioblastoma (GBM) cell line, treated with CX-4945 (5, 10, and 15 μM) for 24 h. Similarly, the hCMEC/D3 cell line was used to mimic the blood–brain barrier (BBB), examining the ability of CX-4945 to restore BBB homeostasis, after stimulation with lipopolysaccharide (LPS) and then treated with CX-4945 (5, 10, and 15 μM). **Results**: We reported that CX-4945 reduced the proliferative activity and modulated the main pathways involved in tumor progression including apoptosis. Furthermore, in confirmation of the in vitro study, performing a xenograft model, we demonstrated that CX-4945 exerted promising antiproliferative effects, also restoring the tight junctions’ expression. **Conclusions**: These new insights into the molecular signaling of CK2 in GBM and BBB demonstrate that CX-4945 could be a promising approach for future GBM therapy, not only in the tumor microenvironment but also at the BBB level.

## Simple Summary

Research for glioblastoma (GBM) treatment is continuously advancing. Of relevance is the use of the casein kinase 2 (CK2) inhibitor. Here, we aimed to investigate the ability of CX-4945, CK2 inhibitor, to counteract tumor aggressiveness, as well as to restore the homeostasis of the blood-brain barrier (BBB) and of tight junctions, through its antiproliferative activity.

## 1. Introduction

Glioblastoma (GBM) is a malignant and aggressive form of cancer among brain tumors, with a low survival rate. Traditional therapeutic approaches, such as surgery, chemotherapy, and radiotherapy, do not produce satisfactory life extension since less than 5% of patients diagnosed with GBM survive for more than 5 years [1]. The GBM microenvironment produces several pro-inflammatory cytokines and mediators, including interleukin-1β (IL-1β) and interleukin-8 (IL-18), that play key roles in the GBM through activation of downstream targets such as protein kinases signaling pathways, Janus kinase/signal transducer, and activator of transcription 3 (JAK/STAT3) [2]. The blood–brain barrier (BBB), mediated by endothelial tight junctions, is altered in glioblastoma, resulting in cerebral edema, but the mechanism underlying BBB breakdown is unknown. During GBM progression, uncontrolled proliferation of cells leads to the instability of the tumor microenvironment with tumor cells also invading surrounding tissue up to the BBB, where results of said invasion are characterized by loss of integrity and breakdown of tight junctions of the endothelium, contributing to the extravasation of the tumoral cells expressing cytokines (interferon-γ (IFN-γ), tumor necrosis factor-α (TNF-α), and growth factors [3]. In this perspective, canonical treatments including radiotherapy and chemotherapy are strongly limited in treating GBM due to toxicity to healthy brain areas that have more impactful consequences leading to brain damage and decreased quality of life in patients; thus, the development of novel pharmacological targets that could show tumor suppressive features is of great importance. In recent studies, casein kinase 2 (CK2), a tetrameric serine/threonine kinase composed of two catalytic subunits (2 α or 2α’) and two non-catalytic β subunits, was found to be overexpressed in GBM samples [4,5]. This was confirmed through analyses such as QPCR and R2 microarray (R2: microarray analysis and visualization platform) where primary samples from GBM patients were analysed, reporting high CK2α protein expression [6]. Phase I/II clinical trials are ongoing on CK2 inhibitors, particularly Silmitasertib (CX4945), whose activity is directed at various solid tumors, and in the context of CNS tumors, it has only been examined in patients with neuroblastoma and medulloblastoma [7]. Physiologically, CK2 is involved in cell cycle, apoptosis, and signal transduction; its inhibition was also reported to decrease cell migration and adhesion, increase the apoptotic process, and inhibit tumor growth of GBM in both in vitro and in vivo GBM xenograft models [5,8,9]. CK2 directly orchestrates varied cellular signaling pathways: it potentiates and modulates phosphatidyl inositol 3-kinase/protein B (PI3K/Akt), induces the degradation of IκB-α by reducing its inhibitory action, and controls the JAK/STAT3 pathway [10]. Taking advantage of the wide spectrum of regulated pathways that CK2 drives, it could be considered a drug target. CX-4945 (Silmitasertib) has been designed as a potent ATP-competitive CK2 inhibitor of subunits 2α and 2α’ [11], with excellent pharmacological properties against tumor progression. Silmitasertib is in human trials for various forms of tumor, including multiple myeloma [12] and renal carcinoma [13,14], and is indicated by the FDA as an “orphan drug” for cholangiocarcinoma. Thus, this study aims to investigate whether CK2 plays a role in the GBM-induced inflammatory JAK/STAT pathway, oxidative stress, and angiogenesis by using its inhibitor CX-4945 in U-87 and endothelial cells, respectively. Furthermore, through the GBM xenograft model, the antiproliferative role of CX-4945 will be investigated, providing further evidence that CK2 inhibition could influence the GBM microenvironment also restoring the BBB stability directly involved in brain tumor progression and thus reducing the tumorigenic power of GBM.

## 2. Materials and Methods

### 2.1. Materials

CX-4945 (brand name Silmitasertib) was purchased from Selleckchem (Rome, Italy) (Cat# HY-50855/CS-0563; MCE^®^). CX-4945 dissolved in dimethyl sulfoxide (DMSO), following manufacturer’s instructions. LPS from *E. coli* (0111:B4) was obtained from Sigma-Aldrich (St. Louis MO, USA) (Cat# L3012-5MG). LPS was dissolved in basal medium (1 mg/mL, stock solution).

### 2.2. In Vitro Study

#### 2.2.1. Cell Cultures

Different cell lines were used in this study: normal human astrocyte cell lines (Homo sapiens astrocytes NHA-Astrocytes AGM LONZA^®^ CC-2565™) were used as normal counterparts and cultured with astrocyte culture medium and maintained in the incubator at 37 °C with 5% CO_2_. Human GBM cell lines U-87MG (U-87 MG ATCC^®^ HTB-14TM Homo sapiens brain glioblastoma grade IV), U-138MG (U-138 MG ATCC^®^ HTB-16™ Homo sapiens brain glioblastoma grade IV), and A-172 (A-172 ATCC^®^ CRL-1620™ Homo sapiens brain glioblastoma) purchased from ATCC (American Type Culture Collection, Rockville, MD, USA), brain microvascular endothelial cell line (hCMEC/D3) and human blood–brain barrier endothelial cells (BMECs) were cultured as previously described [15,16].

#### 2.2.2. Cell Treatments

NHA and glioblastoma cell lines (U-87, U-138, and A-172 cell lines) were plated on 96-well plates at 4 × 10^4^ cells/well density to a total volume of 150 μL. After 24 h, cells were treated with CX-4945 at different concentrations (1, 5, 10, and 15 μM) [17] and dissolved in the basal medium, for 24 h. In the same way, hCMEC/D3 and BMEC cells were plated on 96-well plates at a density of 4 × 10^4^ cells/well to a final volume of 150 μL. After 24 h, cells were treated with CX-4945 (1, 5, 10, 15, 30, 100, and 300 μM). In another set, cells were treated with LPS, dissolved in basal medium, at a concentration of 10 mg/mL. The same cells were treated the day after with increasing concentrations of CX-4945 (1, 5, 10, and 15 μM) [18] and incubated at 37 °C for 24 h. All of the used concentrations were chosen based on the literature.

#### 2.2.3. Cell Viability Assay (MTT Assay)

After 24 h from treatments with CX-4945, all cells were incubated with 3-(4,5-dimethylthiazol-2-yl)-2,5-diphenyltetrazolium bromide (tetrazolium dye MTT; M2128; Sigma^®^; St. Louis, MO, USA) (0.2 mg/mL) at 37 °C for 60 min; the medium was removed, and cells were lysed with 100 mL DMSO. The amount of MTT reduced to formazan was quantified by measuring the optical density at 540 nm (OD540) with a microplate reader.

#### 2.2.4. Experimental Design

NHA, U-87, U-138 and A-172 cells were plated in different wells and differentially treated as follows:

— NHA cells were maintained with basal medium (AGM);
— U-87, U-138, and A-172 cells were maintained with basal medium (MEM);
— NHA, U-87, U-138, and A-172 cells were treated with CX-4945 1 μM for 24 h;
— NHA, U-87, U-138, and A-172 cells were treated with CX-4945 5 μM for 24 h;
— NHA, U-87, U-138, and A-172 cells were treated with CX-4945 10 μM for 24 h;
— NHA, U-87, U-138, and A-172 cells were treated with CX-4945 15 μM for 24 h.

Also, hCMEC/D3 cells were plated in different wells and differentially treated as follows:

— hCMEC/D3 cells were maintained with basal medium (DMEM);
— hCMEC/D3 cells were treated with LPS 10 μg/mL for 24 h;
— hCMEC/D3 cells were treated with LPS 10 μg/mL for 24 h and then with CX-4945 5 μM for other 24 h;
— hCMEC/D3 cells were treated with LPS 10 μg/mL for 24 h and then with CX-4945 10 μM for other 24 h;
— hCMEC/D3 cells were treated with LPS 10 μg/mL for 24 h and then with CX-4945 15 μM for other 24 h.

#### 2.2.5. Colony Formation Assay in U-87 Cells

The effect of CX-4945 on U-87 cell proliferation was evaluated by performing the colony formation assay [19,20]. U-87 cells were seeded into six-well plates at 10^3^ cells/well density, for a final volume of 2 mL. After 24 h, cells were treated with CX-4945 at different concentrations (5, 10, and 15 μM), dissolved in basal medium, and replaced in the incubator until the formation of sufficiently large colonies. After 24 h of the treatment, cells were rinsed carefully with PBS; subsequently, basal medium with FBS 10% was added, and cells were incubated for 10 days (with a basal medium change enriched with FBS 10%, three times a week). After this period, cells were washed with PBS and then fixed with 4% paraformaldehyde (PFA) for 30 min. Subsequently, cells were stained with 0.1% (*w*/*v*) of crystal violet, and images were taken by using a bright-field microscope.

#### 2.2.6. Western Blot Analysis for Inflammation, Oxidative Stress, Apoptosis, and Angiogenesis Evaluation

Western blot analysis on the U-87 cell line was performed as previously described [21] by using the following antibodies: anti-NF-κB p65 (1:500; Santa Cruz Biotechnology, Dallas, TX, USA; sc-8008); anti-IL-1β (1:500; Santa Cruz biotechnology; Dallas, TX, USA; sc-32294); anti-iNOS (1:500; Santa Cruz Biotechnology; sc-7271; Dallas, TX, USA); anti-COX2 (1:500; Santa Cruz Biotechnology; sc-37686; Dallas, TX, USA1); anti-IL-18 (1:500; Santa Cruz Biotechnology; sc-7954; Dallas, TX, USA), anti-p53 (1:500; Santa Cruz Biotechnology; sc-126; Dallas, TX, USA), anti-BID (1:500; Santa Cruz Biotechnology; sc-11423; Dallas, TX, USA), anti-caspase3 (1:500; Santa Cruz Biotechnology; sc-7272; Dallas, TX, USA), anti-heme oxygenase 1 (HO1) (1:500; Abcam; ab13248; Cambridge, UK), anti-MnSOD (1:500; Millipore; 3542472; Burlington, MA, USA) and anti-Nrf2 (1:500; Santa Cruz Biotechnology; sc-364959; Dallas, TX, USA), anti-NGF (1:1000; Cell Signaling; 2046; Danvers, MA, USA), and anti-neurotrophin 3 (NT-3) (1:500; Santa Cruz Biotechnology; sc-518099; Dallas, TX, USA). For hCMEC/D3 cells, the primary antibodies used were: anti-JAK1 (1:500; Invitrogen; MA5-42649; Waltham, MA, USA), anti-STAT-3 (1:500; Abcam; ab280212; Cambridge, UK), anti-vascular endothelial growth factor (VEGF) (1.500; Santa Cruz Biotechnology; sc-7269; Dallas, TX, USA), anti-CK2α (1:1000, Cell Signaling, 2656; Danvers, MA, USA), anti-pCK2α (1:1000, cat.8738), anti-BDNF (1:500 Abcam, ab108319; Cambridge, UK), and anti-GDNF (1:500 Abcam, ab18956; Cambridge, UK). The membranes were then incubated with secondary antibodies (1:2000; Jackson ImmunoResearch, West Grove, PA, USA) for 1 h. Anti-β-actin antibody (1:500; Santa Cruz Biotechnology, Dallas, TX, USA; sc-81178 and sc-8432) was used to confirm equal amounts of protein sample. The densitometric values obtained are normalized to β-actin and expressed as % of the control.

#### 2.2.7. Measurement of Oxidative Product Levels in GBM Cells

To measure intracellular hydrogen peroxide (H_2_O_2_) levels in GBM cells [22], the intracellular hydrogen peroxide assay (#MAK164, Sigma-Aldrich, St. Louis, MO, USA) [23] was performed by following manufacturer’s instructions.

#### 2.2.8. Immunofluorescence of BDNF and GDNF on U-87 Cells, ZO-1 and Occludin on hCMEC/D3 Cells

As previously described, immunofluorescence was performed on U-87 and hCMEC/D3 cells [24]. U-87 and hCMEC/D3 cells were plated on slide 2 × 10^5^ for a final volume of 1 mL and after 24 h were treated with CX-4945 at concentrations of 5, 10, and 15 μM. After 24 h, cells were fixed on the slide with 4% PFA for 15 min and subsequently rinsed with PBS solution for 2 min; the slides were then permeabilized in 0.2% Triton-X/PBS and blocked with 10% FBS/PBS for 10 min. The cells were incubated overnight at 4 °C with primary antibodies, respectively: anti-brain-derived neurotrophic factor (BDNF) (1:100; Santa Cruz Biotechnology; sc-65514), anti-glial cell-derived neurotrophic factor (GDNF) (1:100; Santa Cruz Biotechnology; sc-328), anti-Zonula Occludens-1 (ZO-1) (1:100; Santa Cruz Biotechnology; sc-33725), and anti-occludin (1:100; Santa Cruz Biotechnology; sc-133256). Then, the slides were rinsed gently with PBS solution and incubated with Alexa Fluor-594 antibody (1:1000 *v*/*v* Molecular Probes, Eugene, Oregon, USA) for 1 h at 37 °C. Finally, the cells were washed in PBS solution and for nuclear staining, 4′,6′-diamidino-2-phenylindole (DAPI; Hoechst, Frankfurt; Germany) 2 mg/mL in PBS was added. Sections were observed and photographed at 40× magnification using the Nikon Eclipse Ci-L microscope.

### 2.3. In Vivo Study

#### 2.3.1. Animals

In this study, we used BALB/c nude male mice (25–30 g; 6–8 weeks of age) from Envigo (Milan, Italy). The animals were housed by following animal facility regulation. The animal study was approved by the University of Messina (n° 783/2021-PR) following Italian regulations on the use of animals (D.M.116192) and Directive legislation (EU) (2010/63/EU) amended by Regulation (EU) 2019/1010.

#### 2.3.2. Xenograft Model of GBM

Mice received a subcutaneous injection into the right flank with 3 × 10^6^ U-87 GBM cells in 0.2 mL of PBS and 0.1 mL of Matrigel (BD Bioscience, Bedford, MA, USA) [25]. Then, animals were monitored daily for morbidity and mortality and weighed weekly to assess body weight change as well as general health. When the tumor reached 200–300 mm^3^ (approximately after three weeks), the mice were treated with CX-4945. CX-4945 was orally administered at a dose of 50 mg/kg or 100 mg/kg dissolved in saline for two weeks. The doses of CX-4945 were established based on previous studies [21,26].

Mice were randomly divided in 4 experimental groups:

- Control group (GBM): the animal received only oral administration of saline solution (n = 18);
- GBM + CX-4945 (50 mg/kg): the animal with GBM received administration of CX-4945 at a dose of 50 mg/kg for two weeks (n = 18);
- GBM + CX-4945 (100 mg/kg): the animal with GBM received administration of CX-4945 at a dose of 100 mg/kg for two weeks (n = 18).

Mice were sacrificed five weeks after U-87 GBM cell injection, and the tumor mass was collected and used for further analysis. The minimum number of mice for each experimental group was estimated with the statistical test “ANOVA: Fixed effect, omnibus one-way” with G-power 3.1 software. The G∗ power analysis produced a minimum total number of mice of 54, which, when divided into the 3 groups, leads to an effective sample size of 18 mice/group.

#### 2.3.3. Histological Evaluation

Five weeks after GBM inoculation, subcutaneous tumor masses were excised from the right flank and processed for histological evaluation [21,26]. The following parameters have been considered: 0 = no tumor spread, 1 = poor tumor differentiation, 2 = moderate differentiation and nuclear atypia, 3 = differentiated tumor cells, 4 = well-defined nuclear atypia and spread, 5 = extreme spread. Representative images were taken with a 20× objective lens using a Nikon Eclipse Ci-L microscope.

#### 2.3.4. Immunohistochemistry Analysis for Ki67 and TGF-β Proliferation Markers

Two important proliferative markers in the context of GBM, Ki67, and TGF-β [27], were evaluated by immunohistochemical staining [28,29]. GBM sections were incubated overnight (O/N) using the following primary antibodies: anti-Ki67 (Cat #MA5-14520; 1:100 in PBS, *v*/*v*; Invitrogen; Waltham, MA, USA) as proliferation activity marker and TGF-β as an oncogenic marker. At the end of incubation with the primary antibodies, the sections were washed with PBS and incubated with a secondary antibody (Santa Cruz Biotechnology, Dallas, TX, USA) for 1 h at room temperature (RT). The reaction was revealed via a chromogenic substrate (DAB), and counterstaining with nuclear fast-red was performed. The percentage area of immunoreactivity (determined by the number of positive pixels) was expressed as the % of total tissue area (red staining) within five random fields and analyzed by using a computerized image analysis system (Leica QWin V3, Cambridge, UK). All stained sections were observed and analyzed in a blinded manner at an objective lens of 20x and 40x by using a Nikon Eclipse Ci-L microscope.

### 2.4. Statistical Analysis

All values are given as mean ± standard deviation (SD) of “N” observations. The results were analyzed by one-way analysis of variance (ANOVA) followed by a Bonferroni post hoc test for multiple comparisons. A *p*-value of less than 0.05 was considered significant.

## 3. Results

### 3.1. In Vitro Study

#### 3.1.1. CX-4945 Reduced Viability and Proliferative Activity of Human GBM Cells

**Figure 1:**
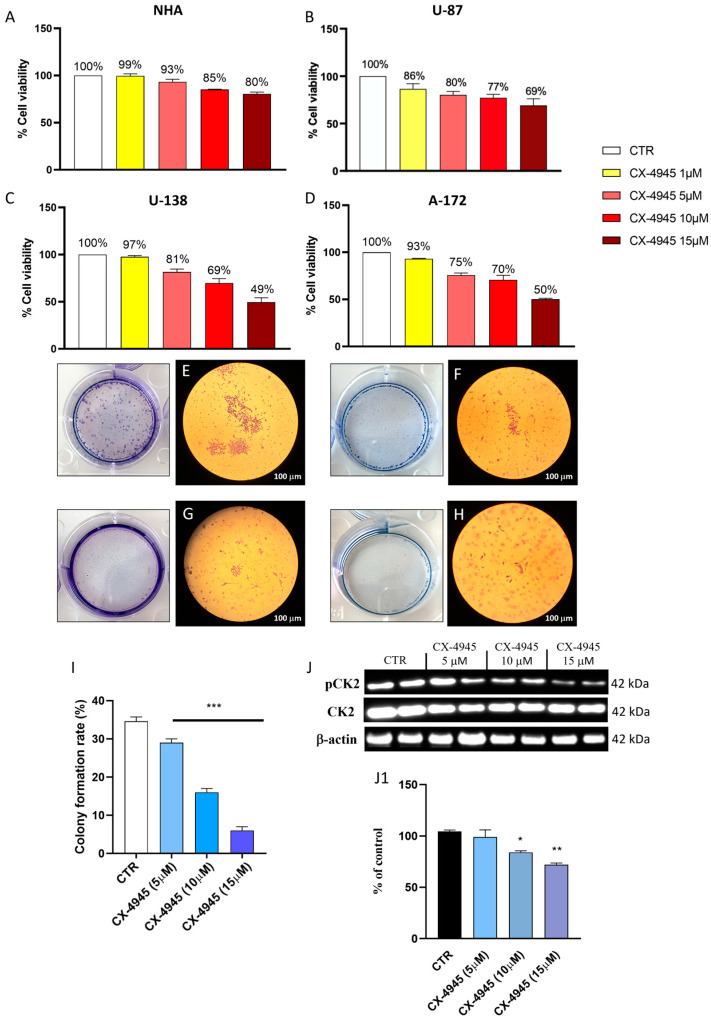
Effect of CX-4945 on viability and proliferative activity of the GBM cells. (**A**) MTT assay detects no toxic effect of CX-4945 in NHA cells, maintaining the viability of these cells. (**B**) CX-4945 treatment significantly reduces U-87 cell viability by around 80–69% at concentrations of 5, 10, and 15 μM. (**C**,**D**) Other GBM cells (U-138 and A-172) show a low percentage of viability after the treatment with CX-4945 at concentrations of 5, 10, and 15 μM. (**E**–**H**) Proliferative activity of U-87 cells blocked by treatment with CX-4945 in a concentration-dependent manner (quantification (**F**)). (**J**), densitometric analysis (**J1**)) Western blot analysis of p-CK2. * *p* < 0.05 vs CTR; ** *p* < 0.01 vs. CTR; *** *p* < 0.001 vs. CTR. (N = 3). (**I**). Colony formation rate. Original western blots are presented in File S1.

The GBM cells are characterized by rapid growth and the invasion of adjacent brain regions [30,31]. Firstly, we evaluated the cytotoxicity of CX-4945 in NHA cells as control [21,32,33] and then we evaluated the effect of CX-4945 on human GBM cells (U-87, U-138, and A-172 cell lines). The results of the MTT assay showed that all CX-4945 concentrations (1, 5, 10, and 15 μM) exerted no cytotoxic effect in NHA cells (Figure 1A). However, CX-4945 reduced U-87, U-138, and A-172 cell viability in a concentration-dependent manner (Figure 1B), especially the concentrations of 5, 10, and 15 μM, which reduced the cell viability of U-87cells to 80%, 77%, and 69%, respectively. While, the same concentration of CX-4945 (5, 10, and 15 μM) reduced the viability of other GBM cells (U-138 and A-172 cell lines) from 81% to 49% (Figure 1C,D). Concentrations of 30, 100, and 300 μM decreased cell viability by around 50–30%, so we did not consider them for further analysis. Also, since the 1 μM concentration reduced the viability to only 86% compared to the control group (Figure 1A), we considered the concentrations of 5, 10, and 15 μM for further analysis. Moreover, since the viability of the GBM cell lines is almost the same, we chose to continue the subsequent analyses using exclusively the U-87 cell line, because this cell line represents one of the most validated cell lines in the field of GBM research [34]. Furthermore, to confirm the ability of the casein kinase 2 inhibitor (CX-4945) to arrest the proliferation of U-87 cells, the colony formation assay was performed (Figure 1E–H, score Figure 1I), and the results obtained demonstrate that CX-4945 significantly counteracted cell proliferation in a concentration-dependent manner (Figure 1E–H, score Figure 1I) compared to the control group (Figure 1E, score Figure 1I). To confirm the inhibitory effect of CX-4945 on CK2, Western blot analysis was performed in cell lysates of U-87 cells; the results demonstrated that CX-4945 at a concentration of 5 µM did not reduce p-CK2 levels compared to CTR cells. Conversely, CX-4945 at the higher concentrations of 10 µM and 15 µM significantly reduced p-CK2 levels compared to CTR cells (Figure 1J, densitometric analysis Figure 1(J1)).

#### 3.1.2. CX-4945 Treatment Reduced the Inflammatory Response by Modulating the NF-κB Pathway and Oxidative Stress in U-87 Cells

**Figure 2:**
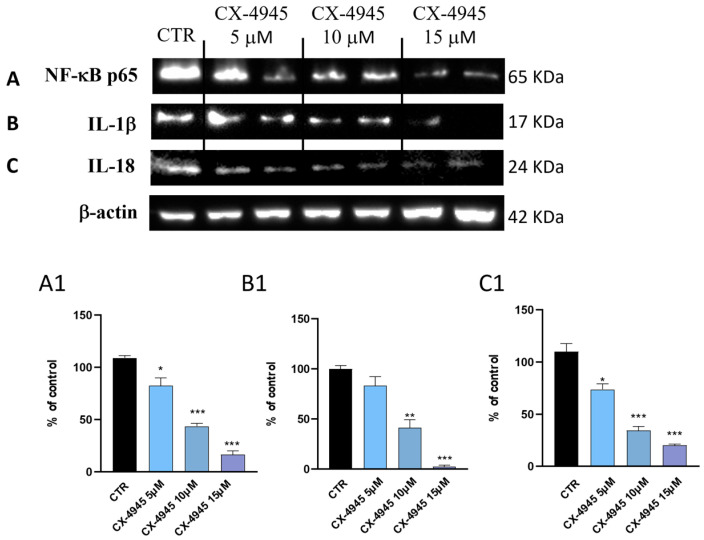
Effect of CX-4945 on the inflammatory cascade. (**A**–**C**) Treatment with CX-4945 at increasing concentrations; 5, 10, and 15 μM, significantly reduced the inflammatory cascade, modulating the NF-κB (score (**A1**)) and the consequent release of pro-inflammatory cytokines, IL-1β (score (**B1**)), IL-18 (score (**C1**)). * *p* < 0.05 vs. CTR; ** *p* < 0.01 vs. CTR; *** *p* < 0.001 vs. CTR. (N = 3). Original western blots are presented in File S1.

GBM is characterized by a high degree of inflammation, which represents an important factor for the maintenance and progression of tumor cells. In this context, NF-κB signaling plays a crucial role in both pre-cancerous and cancer-induced inflammation [35]. It is known that dysregulated NF-κB transcription influences tumor progression and invasiveness [28,36]. Therefore, the effect of CX-4945 on the NF-κB pathway was evaluated on U-87 cell lysates by Western blot analysis (Figure 2A). Our results demonstrated that the protein expression level of NF-κB is much higher in the control group (Figure 2A, densitometric analysis Figure 2(A1)), while CX-4945 treatment for 24 h, at the concentrations of 10 and 15 μM significantly reduced the NF-κB expression level (Figure 2A, densitometric analysis Figure 2(A1)). A lower concentration of CX-4945 (5 μM) did not reduce its expression (Figure 2A, densitometric analysis Figure 2(A1)). However, the reduction of the inflammatory response leads to a reduction in the release of the pro-inflammatory cytokines, such as IL-1β; thus, the concentration of 10 and 15 μM of CX-4945 considerably reduced this expression level (Figure 2B, densitometric analysis Figure 2(B1)). The results also show that the lower concentration of CX-4945 (5 μM) did not reduce IL-1β release in a significant manner (Figure 2B, densitometric analysis Figure 2(B1)). A similar trend has been observed in IL-18 expression level, where IL-18 was significantly reduced by CX-4945 in a concentration-dependent manner (Figure 2C, densitometric analysis Figure 2(C1)).

#### 3.1.3. CX-4945 Modulates Oxidative Product Levels and Apoptosis Pathway in GBM Cells

**Figure 3:**
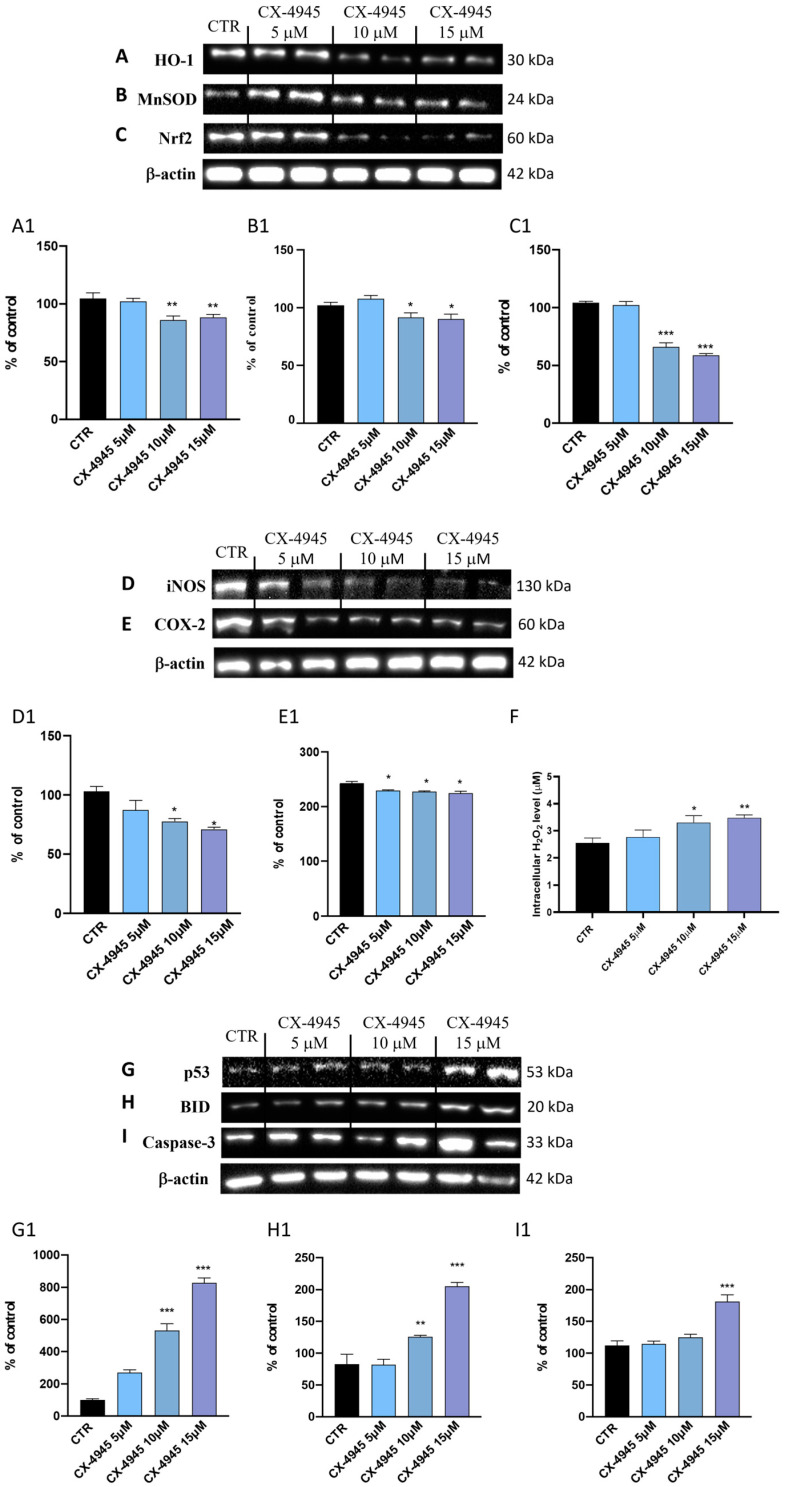
CX-4945 treatment modulated oxidative stress and induced apoptotic processes in U-87 cells. CX-4945 has been shown to increase oxidative stress by modulating HO-1 (score (**A1**)), MnSOD (score (**B1**)), and Nrf2 (score (**C1**)). The consequence of this event led to a decrease of proinflammatory markers iNOS ((**D**); score (**D1**)) and COX-2 ((**E**); score (**E1**)) at 5, 10, and 15 μM concentrations. (**F**) The graph shows the production of intracellular H_2_O_2_ following CX-4945 treatment, in which its levels significantly increased in at both higher concentrations of 10 and 15 μM. (**G**–**I**) Western blot analysis demonstrated that treatment with CX-4945 concentration-dependently induced apoptotic cell death by increasing the expression levels of p53 (score (**G1**)), BID (score (**H1**)), and caspase 3 (score (**I1**)). * *p* < 0.05 vs CTR; ** *p* < 0.01 vs. CTR; *** *p* < 0.001 vs. CTR (N = 3). (**A**) Representative blot of HO-1, (**B**) Representative blot of MnSOD, (**C**) Representative blot of Nrf2. Original western blots are presented in File S1.

Among the various events that characterize the genesis of the tumor, a correlation has been demonstrated between the inflammatory process and the production of reactive oxygen species (ROSs) in tumor cells [37] as tumor cells constantly maintain a balance between the response to oxidative stress and the production of ROS for their survival [38], representing the main mechanisms of toxicity of tumor cells [23]. In this perspective, we evaluated, firstly, the levels of oxidative stress markers (HO-1, MnSOD, and Nrf2) in U-87 cells (Figure 3A–C, densitometric analysis Figure 3(A1–C1)). Our data demonstrated that 24 h after treatment with CX-4945 at the concentrations of 10 and 15 μM, the expression levels of HO-1 and MnSOD significantly reduced compared to the control group (Figure 3A and Figure 3B, densitometric analysis Figure 3(A1) and Figure 3(B1), respectively). As activation of the ROS-p53/Nrf2 signaling pathway is known to induce apoptosis, results from this study revealed that CX-4945 at the higher concentrations of 10 and 15 μM significantly reduced protein expression levels of Nrf2 (Figure 3C, densitometric analysis Figure 3(C1)). Despite that, 5 μM concentration of CX-4945 did not effectively reduce the expression levels of oxidative stress markers (Figure 3A–C, densitometric analysis Figure 3(A1–C1)). In addition, the appearance of oxidative stress in GBM induces the expression of inflammatory cytokines, adhesion molecules, enzymes in the prostaglandin synthesis pathway (such as COX2), and inducible nitric oxide synthase (iNOS) [35]. We also observed that expression levels of pro-inflammatory mediators, such as iNOS and COX-2, were reduced by CX-4945 treatments at all concentrations (5, 10, and 15 μM) (Figure 3D and Figure 3E densitometric analysis Figure 3(D1) and Figure 3(E1), respectively). Finally, recent research reports that if intracellular ROS are produced and controlled, this could be used as a therapeutic tool for tumor treatment. Therefore, to further confirm that CX-4945 modulates ROS production, intracellular H_2_O_2_ levels measured by fluorometric analysis [22,23] revealed a pro-oxidant effect of CX-4945 in inducing subsequent tumor cell death. Compared to the CTR group, in which the H_2_O_2_ concentration is approximately 2–3 μM, CX-4945 in a concentration-dependent manner increased intracellular levels (Figure 3F). The execution of this assay allowed us to confirm further how CX-4945 can modulate ROS, thus counteracting tumor progression. Furthermore, it is known that GBM cells have a high basal metabolic rate [39] and therefore an alteration of the redox balance by anticancer drugs, which induce the increase in ROS production, can direct GBM cells towards a programmed cell death pathway [40,41]. In confirmation of what has just been reported and in support of the reference data in which CK2 inhibition induces apoptosis in tumor cells [42], Western blot analysis performed on U-87 cell lysates in this current investigative study confirmed the ability of CX-4945 to induce apoptosis (Figure 3G–I, densitometric analysis Figure 3(G1–I1)). In particular, treatments with CX-4945 at concentrations of 10 and 15 μM were able to increase the expression levels of pro-apoptotic proteins such as p53 and BID (Figure 3G and Figure 3H, densitometric analysis Figure 3(G1) and Figure 3(H1), respectively) when compared to the control group, which showed a low expression of these proteins (Figure 3G–I, densitometric analysis Figure 3(G1–I1)). However, the lower concentration of 5 μM did not affect the apoptotic process (Figure 3G,H, densitometric analysis Figure 3(G1,H1)). Additionally, the caspase 3 level was increased only by the higher concentration of CX-4945, the 15 μM, 24 h after treatment (Figure 3I, densitometric analysis Figure 3(I1)), when compared to the 5 and 10 μM concentrations, which were not effective in activating apoptosis (Figure 3I, densitometric analysis Figure 3(I1)).

#### 3.1.4. CX-4945 Modulated the Activation of NFs in U-87 Cells

It is known that brain cancer is characterized by uncontrolled cell growth and resistance to cell death, and although NFs are essential for cell growth and differentiation in the CNS [43], and NFs generated by the tumor cells themselves can stimulate tumor growth [29]. In the context of GBM, the most studied neurotrophins are NGF [44] and NT-3 [45], whose interaction with its specific receptor present on the surface of tumor cells, Tropomyosin-related kinase A and C (TrkA) [46] (TrkC), respectively, could contribute to tumor aggressiveness. The use of therapies aimed at inhibiting the NT/Trk signaling pathway is promising to elicit an immune response against cancer [47] reporting how NTs afford differential survival, proliferation, and tumorigenesis.

**Figure 4:**
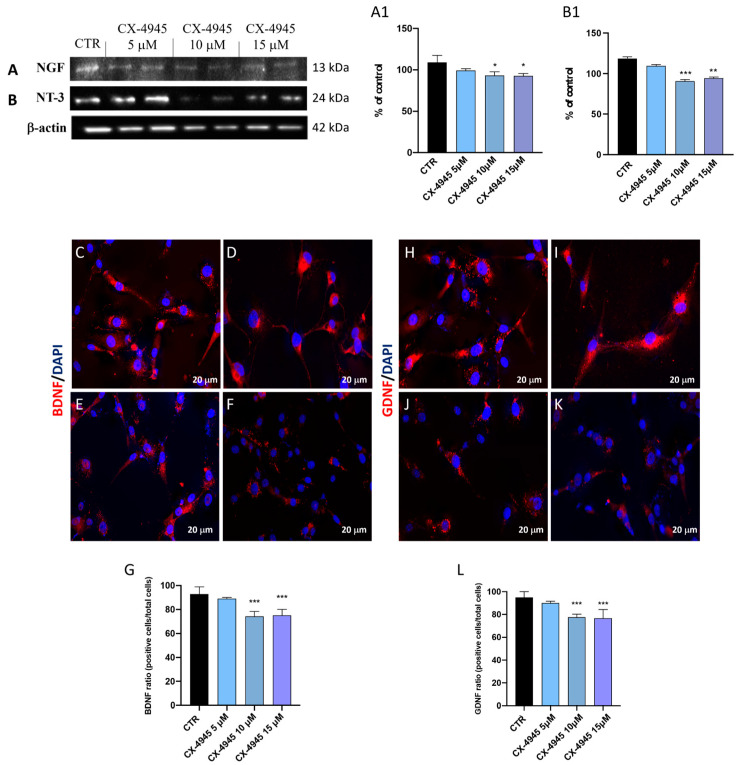
CX-4945 restored the levels of NFs in U-87 cells. Western blot analysis reported the capability of CX-4945, at concentrations of 10 and 15 μM, to modulate the levels of NGF and NT-3 ((**A**,**B**); score (**A1**,**B1**)). (N = 3) Immunofluorescence staining reported positive cells for BDNF and GDNF markers ((**C**–**F**), score (**G**); and (**H**–**K**), score (**L**), respectively). * *p* < 0.05 vs. CTR; ** *p* < 0.01 vs CTR; *** *p* < 0.001 vs. CTR. Original western blots are presented in File S1.

Indeed, the interaction with their specific receptors induces the activation of the MAPK-ERK1/2 pathway promoting the proliferative and migratory activity of glioblastoma cells [48]. This study evaluated the NGF and NT-3 levels by Western blot analysis (Figure 4A and Figure 4B, densitometric analysis Figure 4(A1) and Figure 4(B1), respectively). CX-4945 treatments, at 10 and 15 μM concentrations, reduced the neurotrophic factor NGF levels compared to the control group (Figure 4A and Figure 4B, densitometric analysis Figure 4(A1) and Figure 4(B1), respectively). However, no significant changes have been reported when cells were treated with CX-4945 at the lower concentration of 5 μM. These data have been confirmed by immunofluorescence analysis of BDNF and GDNF (Figure 4C–K, score Figure 4G, and Figure 4L, respectively) where control cells showed strong immunopositivity staining for both BDNF and GDNF (Figure 4C and Figure 4H, scores Figure 4G and Figure 4L, respectively); while the treatment with CX-4945 in a concentration-dependent manner significantly reduced the number of positive cells for both NFs (Figure 4D–F, score Figure 4G; Figure 4I–K, score Figure 4L), suggesting that reduction of BDNF and GDNF in U-87 cells slows down tumor progression.

#### 3.1.5. Evaluation of Cell Viability in hCMEC/D3 and BMEC Cell Lines Following CX-4945 Treatments

**Figure 5:**
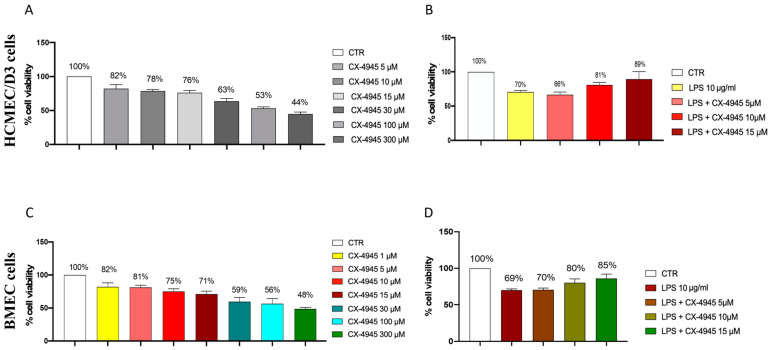
Cell viability assay on hCMEC/D3 and BMEC cell lines. CX-4945 treatments (5, 10, and 15 μM) did not affect cell viability, while concentrations of 30, 100, and 300 μM reduced cell viability of hCMEC/D3 cells (**A**). CX-4945 treatments (5, 10, and 15 μM) increased cell viability after LPS stimulation in hCMEC/D3 cells (**B**). CX-4945 treatments (5, 10, and 15 μM) did not affect cell viability, while concentrations of 30, 100, and 300 μM reduced cell viability of BMEC cells (**C**). CX-4945 treatments (5, 10, and 15 μM) increased cell viability after LPS stimulation in BMEC cells (**D**).

hCMEC/D3 cells were used to perform cell viability assay after treatments with CX-4945. Cells treated with increasing concentrations of CX-4945 (5, 10, 15 μM) showed a good cell viability percentage (around 80 to 70%), while cells treated with higher concentrations of CX-4945 (30, 100, and 300 μM) showed a decreased percentage of cell viability, demonstrating that concentrations higher than 15 μM are cytotoxic to hCMEC/D3 cells (Figure 5A). To study the influence of GBM-secreted factors on human brain endothelial cells, brain microvascular endothelial cells (hCMEC/D3) were used. Based on studies already conducted in in vitro models [49], and to mimic the increased BBB permeability caused by GBM, cells were stimulated with LPS, disrupting the integrity of the BBB and inducing inflammation. The MTT assay revealed how LPS stimulates inflammation and oxidative stress, reducing cell viability by up to 70% (Figure 5A); concurrently, CX-4945 in a concentration-dependent manner protected the cells from further damage induced by LPS as cell viability did not fall below 45%, for the 15 μM concentration of CX-4945 (Figure 5B). Moreover, to confirm that CX-4945 was not cytotoxic at the chosen concentration, we evaluated the cell viability of BMEC cells following CX-4945 treatments and LPS stimulation. Results show that, in BMEC cells, CX-4945 was not cytotoxic at the concentrations of 5, 10, and 15 μM, while increased concentrations up to 300 μM were shown to significantly decrease cell viability (Figure 5C), so we did not consider them for further analysis. When BMEC cells were stimulated with LPS and then treated with CX-4945, the MTT assay revealed how LPS stimulates inflammation, reducing cell viability by up to 70%, while CX-4945 in a concentration-dependent manner (5, 10, and 15 μM) protected cells from LPS-induced damage (Figure 5D).

#### 3.1.6. Treatment with CX-4945 Modulated the JAK1/STAT3 Pathway by Reducing the LPS-Induced Inflammatory Process on HCMEC/D3 Cells

**Figure 6:**
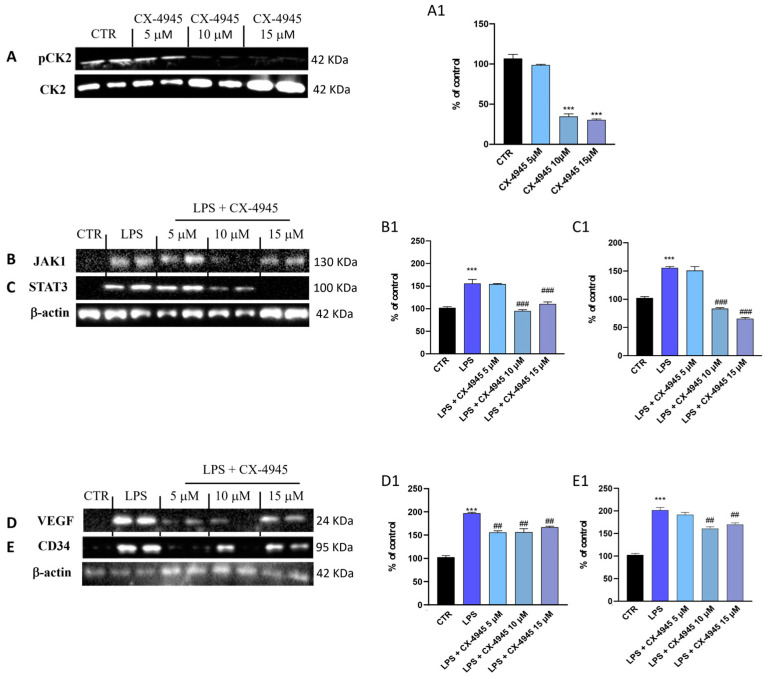
The protective effect of CX-4945 on the epithelial cells damaged by LPS-induced. Western blot analysis of pCK2 in hCMEC/D3 cells ((**A**); score (**A1**)). Western blot analysis on the hCMEC/D3 cell lysates revealed that the LPS group presented a high level of JAK1/STAT3 proteins, while CX-4945 treatment in a concentration-dependent manner reduced the inflammatory response by modulating JAK1/STAT3 pathway ((**B**,**C**), score (**B1**,**C1**)). The angiogenesis was evaluated by Western blot analysis. 24 h after treatment with CX-4946 reduced the angiogenic process ((**D**,**E**), score (**D1**,**E1**)). *** *p* < 0.001 vs. CTR; ## *p* < 0.01 vs. LPS; ### *p* < 0.001 vs. LPS. (N = 3). Original western blots are presented in File S1.

Western blot analysis was performed to investigate CK2 levels in hCMEC/D3 cell lysates after treatment with CX-4945 at concentrations of 5 μM, 10 μM, and 15 μM. The results demonstrated that CX-4945 significantly reduced the p-CK2 levels at higher concentrations of 10 μM and 15 μM compared to untreated hCMEC/D3 cells (Figure 6A, densitometric analysis Figure 6(A1)). Otherwise, the lower concentration of CX-4549 (5 μM) did not significantly reduce p-CK2 levels. JAK/STAT signaling contributes primarily to a wide variety of pro-tumorigenic functions, including proliferation, and anti-apoptosis, by driving aggressive growth, and invasion of cancer cells [50,51]. In this regard, the inflammatory process induced by LPS on epithelial cells first determines the activation of JAK1, which, through phosphorylation, induces the activation of STAT3 [52]. Therefore, active JAK/STAT3 represents signaling upstream of NF-κB to induce the expression of pro-inflammatory cytokines [53]. Results from this study showed that the expression levels of JAK1 were significantly reduced by CX-4945 at the concentration of 10 and 15 µM (Figure 6B, densitometric analysis Figure 6(B1)), compared to LPS-treated cells, while the 5 µM concentration of CX-4945 did not modulate the JAK1/STAT3 pathway (Figure 6B, densitometric analysis Figure 6(B1)). Results also showed that CX-4945 treatment, especially at concentrations of 10 and 15 μM, significantly decreased STAT3 levels (Figure 6C, densitometric analysis Figure 6(C1)) compared to the LPS-treated cells, which showed an increase in STAT3 levels (Figure 6C, densitometric analysis Figure 6(C1)). Meanwhile, 24-h treatment with CX-4945 at a concentration of 5 μM did not modulate the expression level of STAT3 (Figure 6C, densitometric analysis Figure 6(C1)).

#### 3.1.7. CX-4945 Reduced Angiogenesis Following LPS Stimulation in hCMEC/D3 Cells

Among the typical features of GBM tumors, there is an abnormal vascularization through endothelial cells of BBB [54]. Therefore, we evaluated the expression levels of angiogenic markers such as VEGF and CD34 (Figure 6D and Figure 6E, densitometric analysis Figure 6(D1) and Figure 6(E1), respectively). Our study reported that treatment with CX-4945, at the concentrations of 5, 10, and 15 μM significantly reduced VEGF expression levels (Figure 6D, densitometric analysis Figure 6(D1)), compared to the LPS group that triggers an evident pro-angiogenic state (Figure 6D, densitometric analysis Figure 6(D1)). Additionally, 10 and 15 μM concentrations of CX-4945 significantly reduced CD34 expression levels, despite no significant changes observed after treatment with CX-4945 at the lower concentration of 5 μM.

#### 3.1.8. CX-4945 Restores the Integrity of BBB Damaged by LPS Stimulation

**Figure 7:**
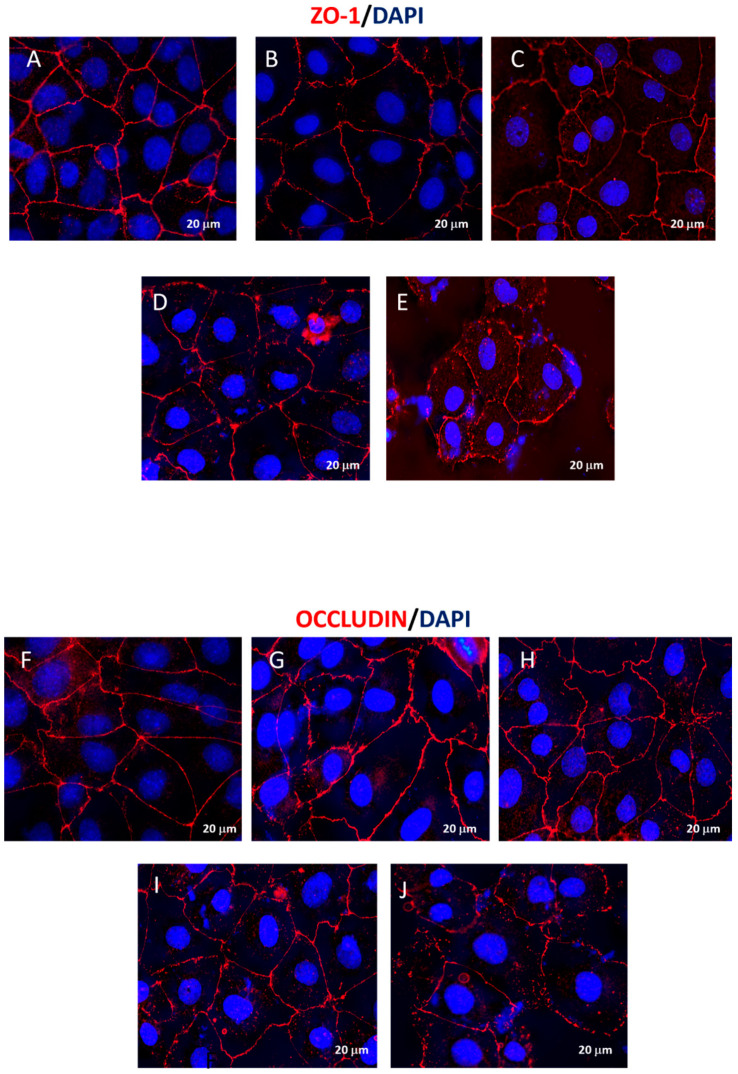
Restoration of the integrity and activity of tight junctions in epithelial cells. Immunofluorescence staining for ZO-1 and Occludin showed a high density of tight junction proteins in the LPS group (**B**,**G**), with a hazy appearance and separation from the cell nucleus, compared to the control group (**A**,**F**). CX-4945 treatment at concentrations of 5, 10, and 15 μM, restored the integrity of the epithelial barrier and the activity of tight junction (**C**–**E**,**H**–**J**).

In GBM, the integrity of the primary tumor mass is influenced by intercellular structures as well as cell-to-cell adhesion, factors that may promote the progression of the tumor mass because of failure in tight junctions (TJs) to suppress cell proliferation [55,56,57]. Immunofluorescence analysis for ZO-1 and Occludin highlights how LPS produced an increasing amount of ZO-1 and Occludin proteins (Figure 7B and Figure 7G, respectively for ZO-1 and Occludin), distributed away from the edges of the brain endothelial cells resulting in a misty appearance in the cytoplasm in comparison with control cells (Figure 7A and Figure 7F, respectively, for ZO-1 and Occludin). Treatments with CX-4945, in a concentration-dependent manner, restored the appearance of TJs (Figure 7C–E; and Figure 7H–J, respectively, for ZO-1 and Occludin).

### 3.2. In Vivo Study

#### 3.2.1. CX-4945 Reduced the GBM Growth in Xenograft GBM Model

**Figure 8:**
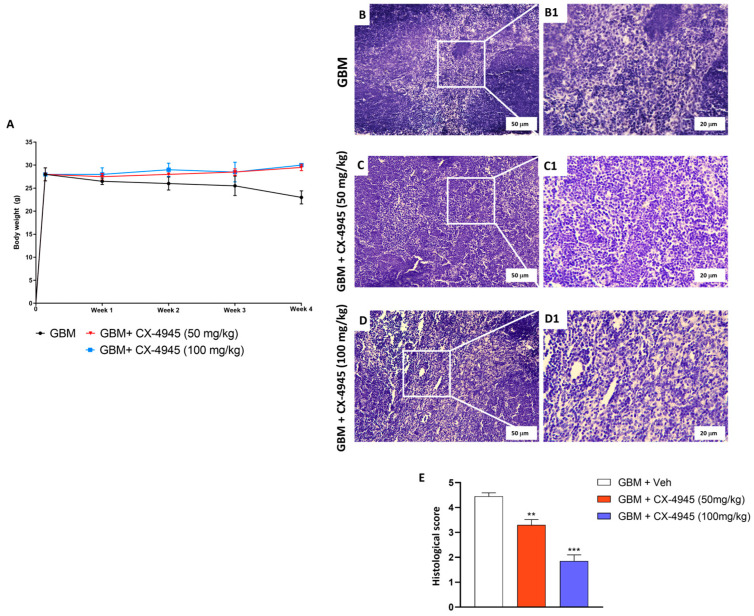
Effect of CX-4945 treatment on growth tumor mass and in xenograft GBM model. (**A**) Representative graph of body weight of mice with GBM cells inoculation. From time 0 until four weeks, untreated mice reported a decrease in body weight (black line) compared with groups of mice who received CX-4945 treatment at the doses of 50 and 100 mg/kg, in which their body weight did not change (lines red and blue, respectively). ((**B**,**B1**) and score (**E**)). Histological image of GBM mice without CX-4945 treatment. ((**C**,**C1**); (**D**,**D1**) (**E**)) Histological score shows treatments with CX-4945, at the doses of 50 and 100 mg/kg, reduced subcutaneous tumor mass as well as neutrophil infiltration compared to GBM mice. Sections were observed and photographed at 20× and 40× magnification. ** *p* < 0.01 vs. GBM; *** *p* < 0.001 vs. GBM.

To confirm data obtained with the in vitro study, the GBM xenograft model was performed [58]. From the day of GBM cells inoculation and subsequent treatment with CX-4945 (doses of 50 and 100 mg/kg), no significant variations in the weight of the mice were recorded (from day 0 to four weeks) (Figure 8A,E). Also, histological analysis of tumor tissue revealed that, in GBM control mice, the subcutaneous tumor mass presented necrotic areas, characterized by homogeneous groups of dead or coalescent cells, and neutrophils (Figure 8B,E). CX-4945 treatments (50 and 100 mg/kg doses) attenuated and reduced the pathological features of GBM (Figure 8 C–E).

#### 3.2.2. The Antiproliferative Effect of CX-4945 on Ki-67 and TGF-β

**Figure 9:**
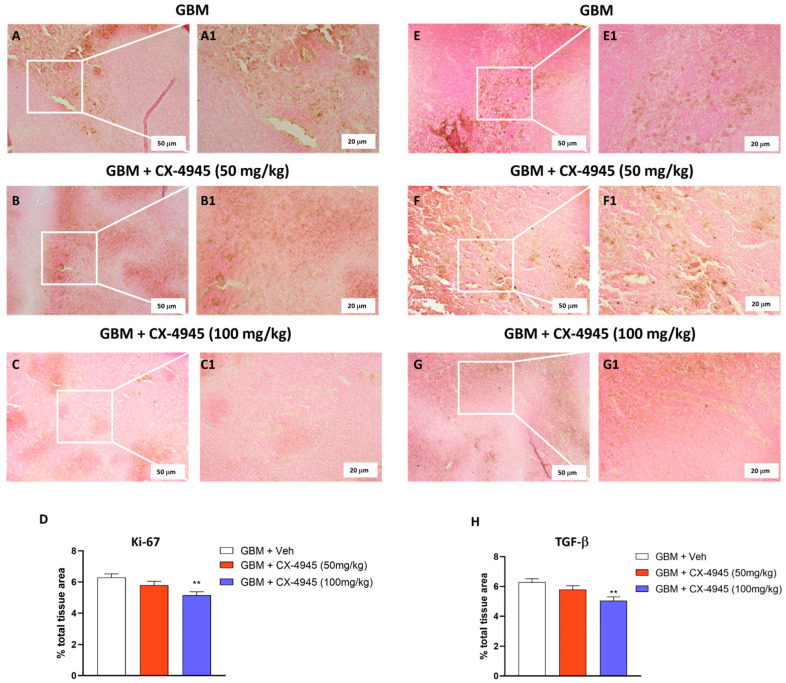
CX-4945 treatment reduced the immunopositivity for proliferative markers, Ki-67 and TGF-β. Ki-67-positive cells were evaluated by immunohistochemical analysis. ((**A**,**A1**) and score (**D**)) mice with GBM show a high number of Ki-67 immunopositivity. ((**B**,**B1**,**C**,**C1**) and score (**D**)) represented mice with GBM whose received CX-4945 treatment at the doses of 50 and 100 mg/kg. The low dose (50 mg/kg) did not significantly reduce the expression levels of proliferative marker Ki-67 more than the high dose 100 mg/kg. ((**E**,**E1**) and score (**H**)) In the same way, TGF-β-positive cells were elevated in the GBM group, while ((**F**,**F1**) and score (**H**)) the treatment with CX-4945 at low dose of 50 mg/kg slightly reduced immunopositivity for TGF-β compared with the dose of 100 mg/kg ((**G**,**G1**) and score (**H**)), which reduced the number of cells positive for TGF-β. Sections were observed and photographed at 20× and 40× magnification. ** *p* < 0.01 vs. GBM.

The antiproliferative effect of CX-4945 on GBM tumor mass was evaluated by immunohistochemical analysis. As previously mentioned, Ki-67 is a nuclear protein associated with cell proliferation, representing an important prognostic marker for the treatment of GBM [59]. The results obtained reported a marked number of Ki-67 positive cells in the GBM mice (Figure 9A,A1 and score Figure 9D). Treatment of CX-4945 at the lower dose of 50 mg/kg did not significantly reduce immunopositivity for Ki-67 marker (Figure 9B,B1 and score Figure 9D), whereas the higher dose of 100 mg/kg of CX-4945 demonstrated a notable capacity to reduce the number of cells positive for the proliferative marker Ki-67 (Figure 9C,C1 and score Figure 9D) compared to the GBM untreated mice. Also, the antiproliferative effect of CX-4945 was examined by evaluating TGF-β expression [60]. GBM untreated mice showed a high immunopositivity for TGF-β (Figure 9E,E1 and score Figure 9H). As observed, treatment with CX-4945, at the higher dose of 100 mg/kg, was able to reduce immunopositivity for TGF-β (Figure 9G,G1 and score Figure 9H) despite no significant changes observed following CX-4945 50 mg/kg administration (Figure 9F,F1 and score Figure 9H).

## 4. Discussion

The U-87 cell line is the most representative glioma cell line, exhibiting the characteristics of human glioblastoma, which includes proliferation and invasion [61]. The results of the present study demonstrated that CX-4945 displayed significant inhibition of proliferation on the U-87 cell line, indicating that U-87 cells might be sensitive to CX-4945 in terms of downregulation of CK2. MTT assay findings from this study demonstrated that CK2 inhibition avoids GBM cell proliferation, a result that is complemented by the colony formation assay showing that CX-4945 strongly suppresses the colony growth of glioma U-87 cells in a concentration-dependent manner. Apoptosis is the most recognized form of cell death in both physiological and pathological status. At present, drug- and molecule-induced apoptosis are the golden strategy for treating tumors including GBM. Numerous other previous studies have shown that CK2 inhibition reduces the proliferation of cancer cells in leukemia or lymphomas [62]; however, the induction of apoptosis by CX-4945 in U-87 cells has yet to be elucidated. In the present study, CX-4945 treatment led to increased apoptosis in human U-87 glioma cells, as measured by proapoptotic proteins p53, BID, and caspase 3, implying that CK2 inhibition exerts significant cytotoxic effects. The increase in proapoptotic protein expression levels suggests an apoptotic mechanism of cell death induced by CX-4945, a mechanism that is probably due to increased protein synthesis or decreased protein turnover mediated through control of multiple targets, including inflammatory pathway of NF-κB and oxidative stress driven by Nrf-2. It has been established in multiple previous studies that CK2 regulates transcription factors such as NF-κB and Nrf-2, reporting a key role of CK2 in inflammation associated with cancer [63]. Previous findings also indicate that many anti-cancer drugs reduce inflammation or oxidative stress in cancer cells, pathways that are crucial to be targeted for exerting anti-survival or pro-apoptotic effects [64]. In this study, when U-87 cells were treated with CX-4945, the NF-κB pathway was reduced, and CK2 inhibition resulted in a decrease of pro-inflammatory mediators involved in the signaling of the transcription factor, leading to the lower expression of downstream ILs such as IL-18 and mediators like iNOS and COX2. This finding reasserts the efficacy of CK2 inhibitor CX-4945 as an anti-cancer and inflammatory agent against GBM. In GBM, cancer cells develop alternative mechanisms to avoid cell death and induce a high proliferation rate essentially through variations in the expression of oncogenes. In parallel, U-87 cells showed an impairment of the redox homeostasis that increases ROS production. Indeed, the “oxygen economy” imbalance in GBM promotes tumor growth, differentiation, and survival of cellular components [41]. Following the development of hypoxia, despite the high ROS production, tumor cells survive in that tumor microenvironment acquiring cell resistance mechanisms and inhibiting the antioxidant system as those of SOD and catalase. To confirm these observations, we evaluated the oxidative stress in U-87 cells, a cell line usually utilized as an experimental standard for founding novel therapeutic strategies for GBM, and we found that CX-4945 exerts its pharmacological effect by downregulating antioxidant enzymes HO-1 and MnSOD; that result aligns with previous findings indicating that both play a significant role in controlling cell proliferation and cell cycle progression. Considering that the phosphorylation of Nrf-2 for the nuclear translocation by CK2, a major kinase for the said phosphorylation, activates transcription of neuroblastoma cells [65], we observed that the CX-4945 inhibitor developed a potential therapeutic effect in human glioblastoma cells by decreasing Nrf-2 translocation into the nucleus and thus providing novel insight into the molecular events of Nrf-2 regulation via CK2 phosphorylation. Since brain cancer is characterized by uncontrolled cell growth, strong evidence demonstrates an elevated level of NFs in GBM, although physiologically they are involved in the induction of neurite outgrowth [66]. Indeed, in many human GBM cells (such as U-87), NGF exerts a mitogenic effect through the interaction with its receptor Trk, localized on the surface of tumor cells [66,67]. Similarly, other NFs such as GDNF promote their growth when released by tumor cells [68].

Furthermore, knowing that CK2 controls NFs by the direct activation of cascade transduction signaling and BDNF or NGF released by glioma cells promoting tumor growth depending on the presence of microglia [68,69], we found a link that exists between neurotrophins-induced signals and regulation of CK2. Accordingly, we showed that by using CK2 inhibitor CX-4945, NGF- and NT-3-mediated neuroprotective signal in GBM most likely leads to the inhibition of phosphorylation activity of CK2. Additionally, the protective effect of both BDNF and GDNF observed in this work seems to be mediated by the activation of CK2 in U-87 cells and reversed by inhibition of its signaling with a specific CK2 inhibitor that decreased the survival of neurotrophins, suggesting that the blocking of GBM progression occurred by CX-4945 required CK2 inhibition of phosphorylation mediated by neurotrophins activation. In the case of brain tumors such as GBM, the new environment induces modifications of the physical and metabolic properties of the BBB, which is known as the blood–brain tumor barrier (BTB). Indeed, the capacity of U-87 cells to migrate along blood vessels, where they are rapidly inserted within the endothelial cells (EC), temporarily pausing or arresting along vascular branch points to proliferate, has been demonstrated [70]. In this study, we characterized ECs viability following LPS-induced damage that mimics disruption of BTB in the GBM environment. As expected, ECs stimulated with LPS proliferated at a lower rate than non-treated ECs, and this low proliferation was maintained by CX-4945 treatments in a concentration-dependent manner. This preliminary result in ECs was of fundamental basis for further evaluation of the JAK1/STAT3 pathway, which is mainly correlated to the phosphorylation activity of CK2. Indeed, CK2 is known to activate the JAK/STAT signaling pathway via interaction with the JAK1 protein that mediates the phosphorylation of STAT3 at Tyr-705 residue [71]. Supporting the literature, we found that the inhibition of CK2 activation by CX-4945 exhibits its anti-oncogenic role and also a converse relation to JAK1 and STAT3 expression levels. As expected, upon overexpression following LPS stimulation, CK2 inhibition decreased JAK1 and STAT3 transcriptional activity as a result of reduced phosphorylation of both. The positive relationship between CK2 and JAK/STAT3 pathway has been assessed here by using CK2 inhibitor whose pharmacological activity is the inhibition of dependent phosphorylation on JAK1 and STAT3 proteins. The exchange of molecules and information via ECs plays a critical role in GBM progression and tumor angiogenesis, which may involve a reprogramming of ECs turnover and stability of BTB. To further elucidate angiogenic pathways in GBM, we here examine the anti-angiogenic effect of CX-4945 in GBM that is likely to be mediated by compensatory activation of pro-angiogenic pathways between tumor cells and ECs. In this context, VEGF and CD34 remain the key molecules thought to drive ECs growth, migration, vessel dilation, and permeability [72]. The inhibition of CK2 led to decreased expression levels of VEGF and CD34 in ECs cells treated with CX 4945. The BBB restricts the free leakage of most large molecules and more than 98% of small-molecule pharmaceuticals, being a nightmare for diagnosis and therapy of CNS diseases and of GBM. In BBB, ECs are closely associated with tight junctions (TJs) belonging to a family of proteins that preserve CNS and maintain neuronal homeostasis. In GBM, differently from normal brain capillaries, the TJs between endothelial cells are damaged for tumor growth and this results in the pathological fenestration and leakage of the BBB [73]. We found the BTB was disrupted to LPS and that lead to a cytoarchitectural translocation of the tight junction proteins ZO-1 and occluding, with CX-4945 treatments restoring an increased amount of ZO-1 and Occludin proteins distributed within the edges of the brain endothelial cells resulting in a clear appearance in the cytoplasm. Further studies on the antitumor effect of CX-4945 through CK2 inhibition were performed by using a GBM xenograft model. CX-4945 at doses of 50 and 100 mg/kg reduced tumor appearance, compared to a GBM mice, where tumor mass was characterized by high neutrophil infiltration and fibrotic scar tissue. As previously mentioned, CK2 promotes GBM cell adhesion, migration, and proliferation, through the activation of several signaling pathways (NF-κB, JAK/STAT, and PI3K/Akt). These events are also controlled and driven by specific markers, such as Ki-67 and TGF-β, that are considered prognostic markers in the GBM filed. Ki-67 expression is upregulated with the increase of the WHO grade [74,75], as well as that of TGF-β, whose concentration levels are high also in the tumor microenvironment [27,76]. In our hands, CX-4945 reduced the invasion of tumor cells and therefore the proliferative capacity of GBM cells, observable by the low number of cells positive for both Ki-67 and TGF-β. The arrest of proliferation and invasiveness, typical of GBM cells, indicates how CX-4945, through the inhibition of its specific target CK2, was able to reduce the aggressiveness of GBM.

## 5. Conclusions

The GBM landscape is extremely complex, and, despite all the new advances, further studies are needed to fully interpret the interactions between the various BTB cell populations and tumor cells, as well as their specifically involved signaling pathways. By exploiting the role of CK2 in GBM and its related signaling pathways (PI3K/Akt, JAK/STAT, and proliferation pathway, as well as regulation of NFs signaling), which contribute to tumorigenesis, these data allowed us to recognize in CX-4945 a promising agent in counteracting GBM. Therefore, these data allowed a point for new prospective aiming to arrest tumor progression by inhibition of the CK2 signaling pathway. However, although this study revealed encouraging results, there are still a few limitations that need to be resolved in the future as the fact that human tumors, especially GBM, are not always possible to translate in preclinical models and that CK2 inhibition could be targeted from different closest parts among the multiple points in its pathway.

## Data Availability

The data presented in this study are available on request from the corresponding author.

## Supplementary Materials

The following supporting information can be downloaded at: https://www.mdpi.com/article/10.3390/cancers16233936/s1, File S1: Original western blots.
