# Supplementary material for: Casein Kinase 2 Inhibitor, CX-4945, Induces Apoptosis and Restores Blood-Brain Barrier Homeostasis in In Vitro and In Vivo Models of Glioblastoma
Source: Cancers (Basel). 2024 Nov 24;16(23):3936. doi: 10.3390/cancers16233936 (PMC11640555; doi:10.3390/cancers16233936)
Supplement: Supplementary file 1 [file cancers-16-03936-s001.zip › cancers-3242339-supplementary.pdf]

Original blots Figure S1

Panel J

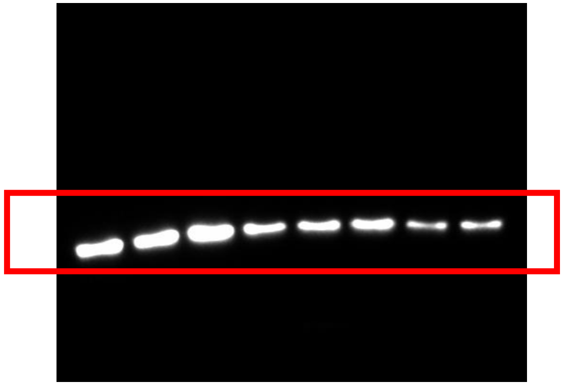

42 KDa

CK2 panel J

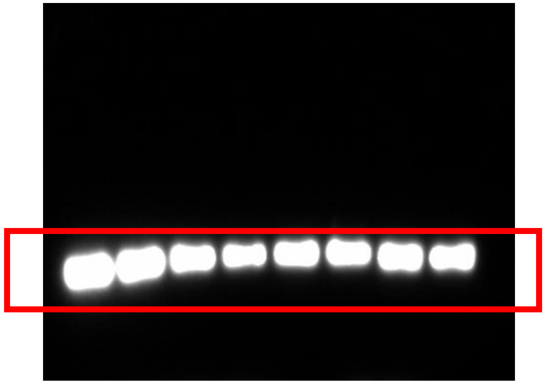

42 KDa

β-actin panel J

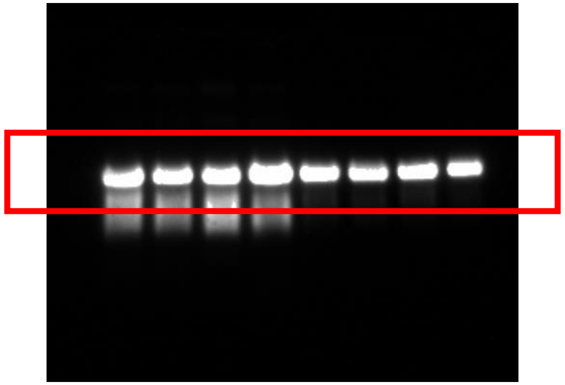

42 KDa

Original blots Figure S2

Panel A

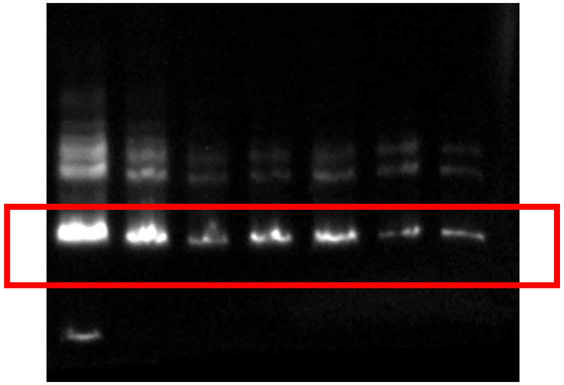

65 KDa

Panel B

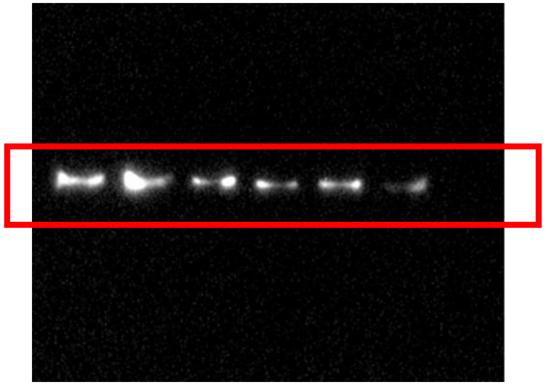

17 KDa

Panel C

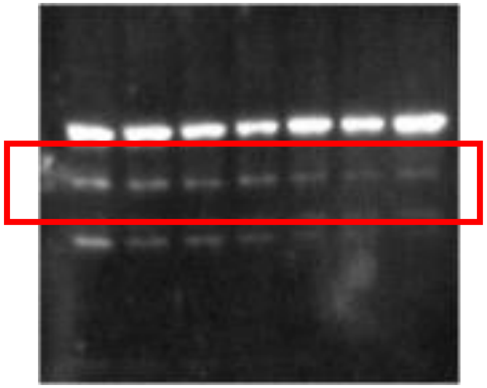

24 KDa

β-actin panels A, B, C

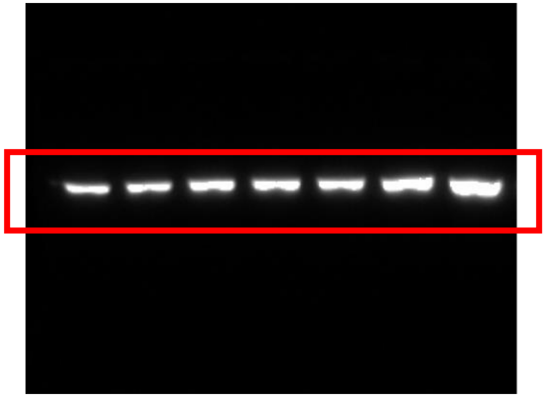

42 KDa

Original blots Figure S3

Panel A

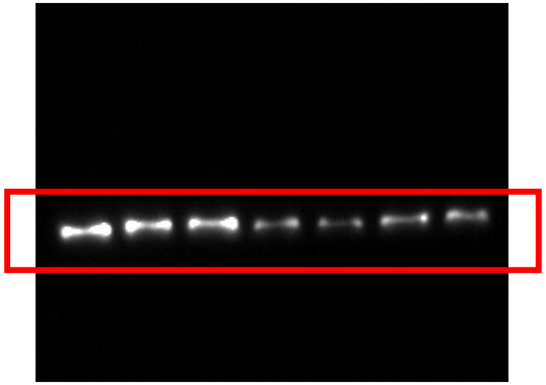

Panel B

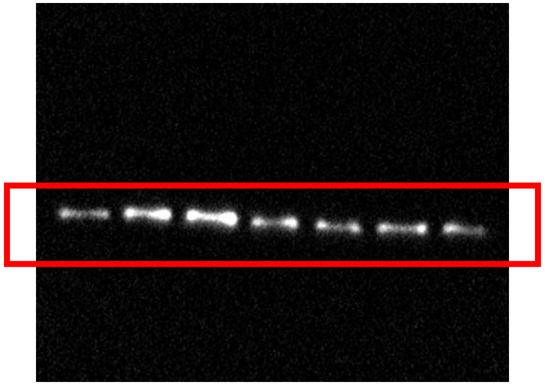

Panel C

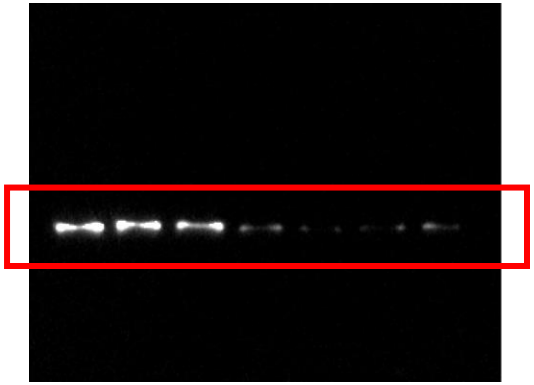

β-actin panels A, B, C

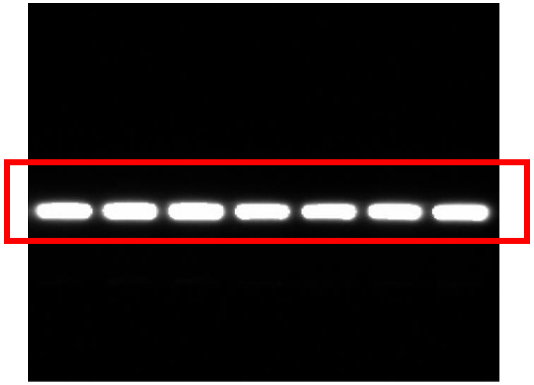

Original blots Figure S3

Panel D

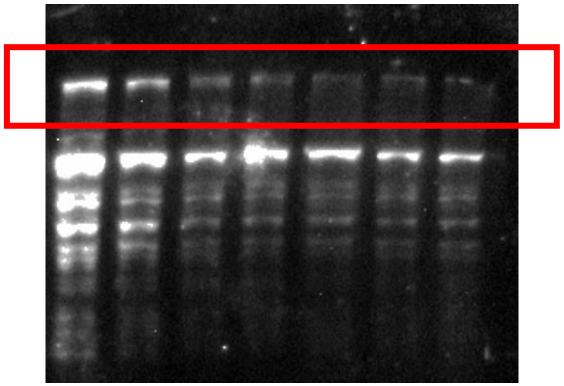

120 KDa

Panel E

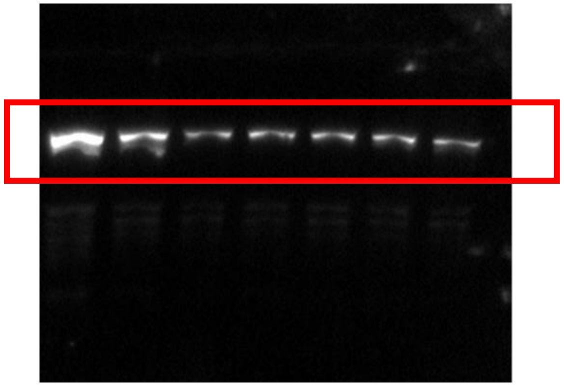

70 KDa

β-actin panels D and E

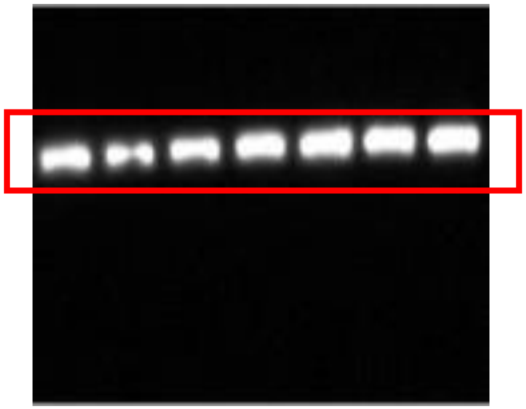

42 KDa

Original blots Figure S3

Panel G

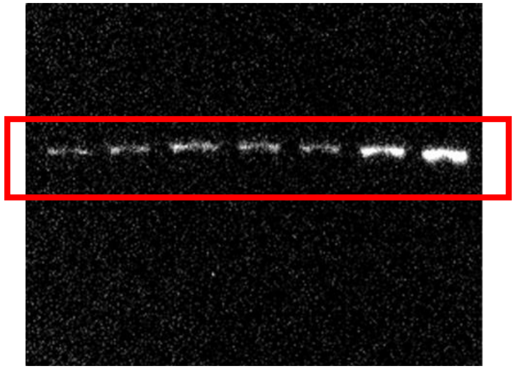

53 KDa

Panel H

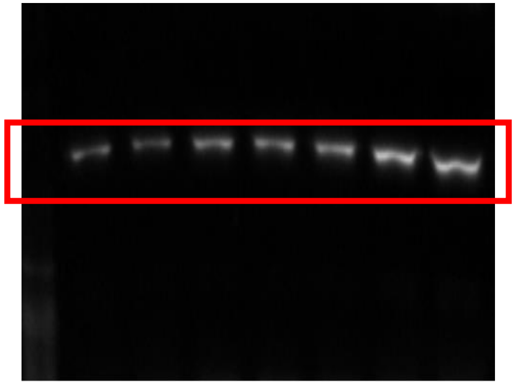

20 KDa

Panel I

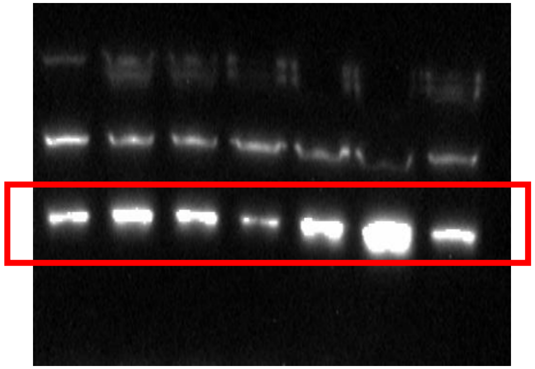

30 KDa

β-actin panels G, H, I

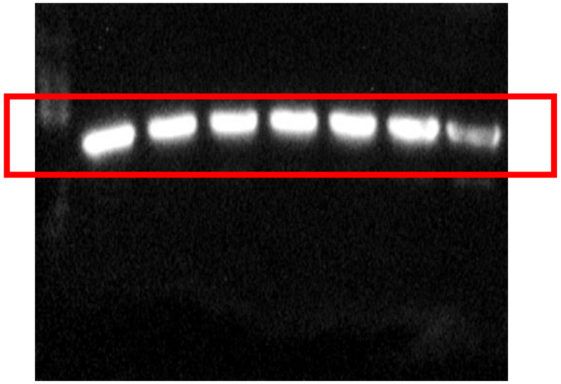

42 KDa

Original blots Figure S4

Panel A

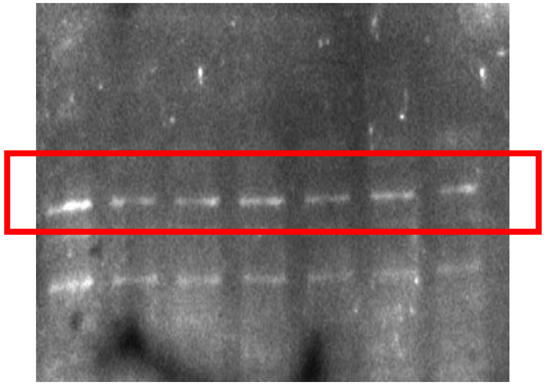

13 KDa

Panel B

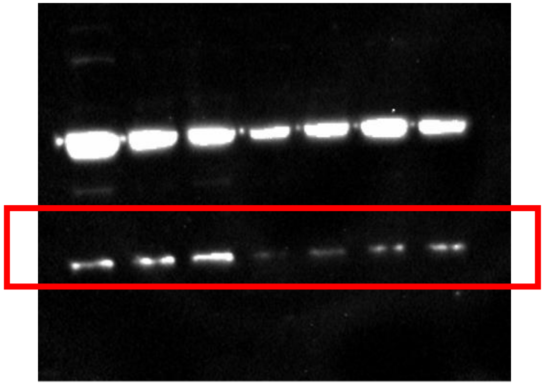

24 KDa

β-actin panels A, B

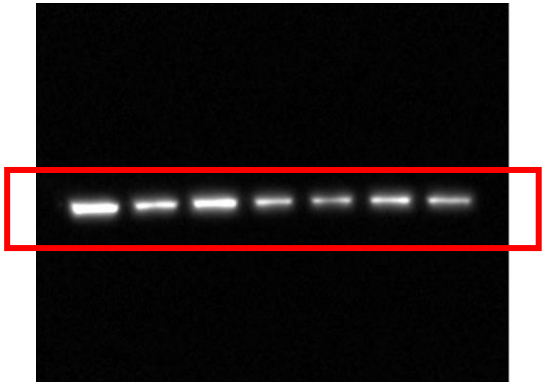

42 KDa

Original blots Figure S5

Panel A

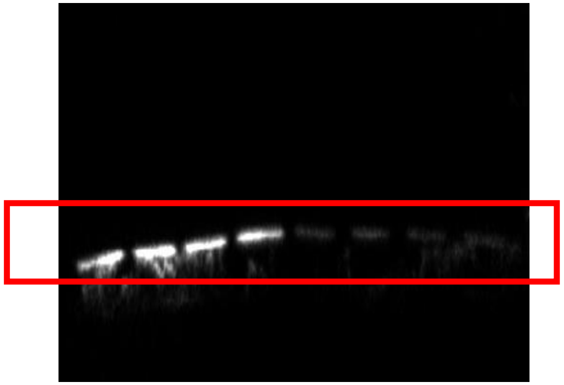

CK2 panel A

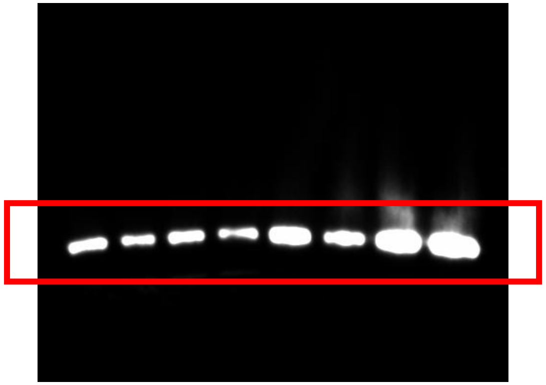

Original blots Figure S5

Panel B

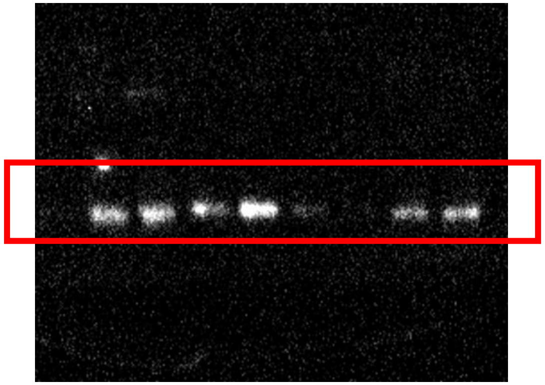

130 KDa

Panel C

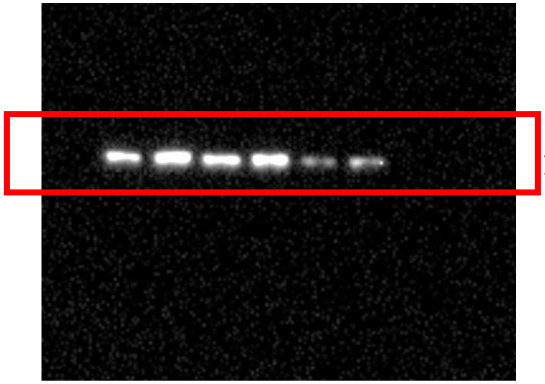

100 KDa

β-actin panels B, C

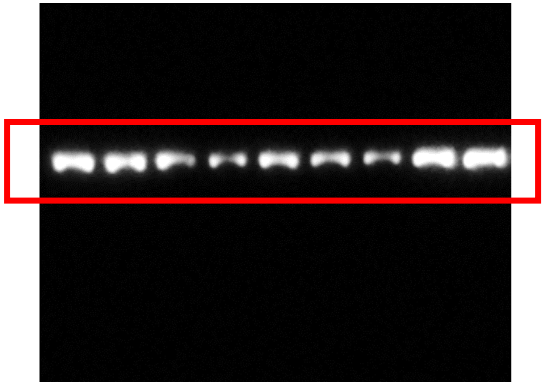

42 KDa

Original blots Figure S5

Panel D

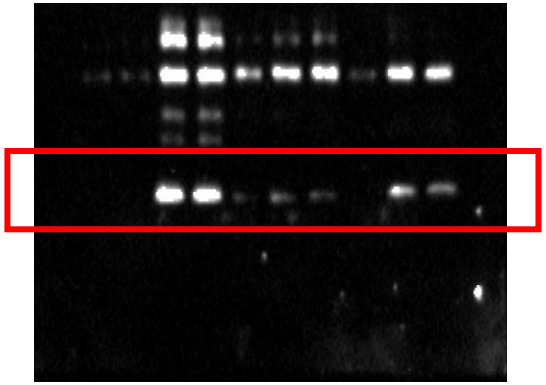

24 KDa

Panel E

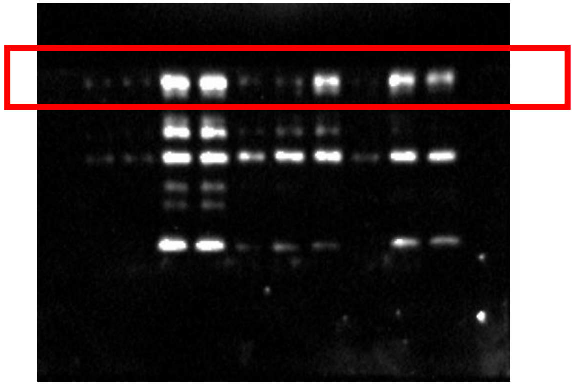

95 KDa

β-actin panels D, E

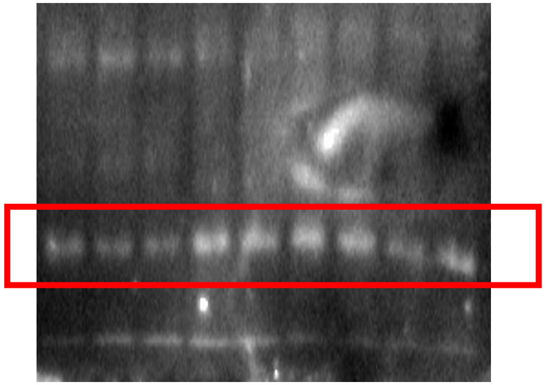

42 KDa
